# LTB4 and montelukast in transplantation-related bronchiolitis obliterans in rats

**DOI:** 10.1186/s13019-017-0605-5

**Published:** 2017-05-25

**Authors:** Zheng-Liang Tu, Zhen-Yu Zhou, Hai-Chao Xu, Jin-Lin Cao, Peng Ye, Lu-Ming Wang, Wang Lv, Jian Hu

**Affiliations:** Department of Thoracic Surgery, the First Affiliated Hospital, College of Medicine, Zhejiang University, NO. 79 Qingchun Road, Hangzhou, 310003 China

**Keywords:** Montelukast, LTB4, Trachea transplantation, Lung transplantation, Bronchiolitis obliterans

## Abstract

**Background:**

Lung transplantation is the only effective treatment for end-stage lung diseases. Bronchiolitis obliterans, which is known as non-infectious chronic lung allograft dysfunction (CLAD) in the new classification, is the greatest threat to long-term survival after lung transplantation. This study investigated the role of leukotriene B4 (LTB4) and montelukast in transplantation-related bronchiolitis obliterans and discussed the pathophysiological significance of LTB4 in chronic rejection.

**Methods:**

Rats were randomly divided into an experimental group (montelukast), a positive control group (dexamethasone), and a blank control group (normal saline solution; NS). Each piece of trachea removed from a F344 rat was transplanted into a Lewis rat through a 5-mm incision at the episternum by subcutaneous embedding. The recipients were treated with gastric lavage with 3 mg/kg · d montelukast suspension, 1 mg/kg · d dexamethasone, and 1 mL/kg · d NS, respectively, in each group. On Day 28, peripheral blood was drawn to measure the white blood cell counts and plasma LTB4 levels. The donor specimens were stained by H-E and Masson, and their organizational structure and extent of fibrosis were visually assessed. The measurement data were compared using one-way analysis of variance, and the categorical data were compared using the chi-square test. A *P* value of less than 0.05 was considered to indicate statistical significance.

**Results:**

The white blood cell counts of the montelukast, dexamethasone, and NS groups were (16.0 ± 4.2) × 10^9^/L, (19.5 ± 11.6) × 10^9^/L, and (25.8 ± 3.6) × 10^9^/L; no statistical significance was found (*P* = 0.101). The concentrations of LTB4 were 2230 ± 592 pg/mL, 1961 ± 922 pg/mL, and 3764 ± 1169 pg/mL, and statistical significance was found between the NS group and each of the others (*P* = 0.009). The percentages of tracheal occlusion were 73.6% ± 13.8%, 23.4% ± 3.2%, and 89.9% ± 11.3%, and statistical significance was found among the three groups (*P* = 0.000).

**Conclusions:**

The study established a model to simulate bronchiolitis obliterans after clinical lung transplantation. Oral administration of montelukast reduced plasma LTB4 levels in rats and played a preventive role against tracheal fibrosis after transplantation. This suggests that LTB4 may be involved in bronchiolitis obliterans after pulmonary transplantation. This study indicates a new direction for research into the prevention and treatment of bronchiolitis obliterans after lung transplantation.

## Background

Lung transplantation is the only effective treatment for end-stage lung diseases [1]. Bronchiolitis obliterans, the most common outcome of chronic rejection after transplantation, has the greatest negative effect on long-term survival after lung transplantation [2, 3]. Studies have shown that occlusion of the airway caused by fibroplasia is the main pathological manifestation of bronchiolitis obliterans and that heterotopic transplantation in animals is a suitable model for the study of this syndrome [4, 5].

Leukotriene B4 (LTB4) is a key inflammatory factor inducing the chemotaxis and activation of polymorphonuclear leukocyte (PMN) [6, 7]. LTB4 mediates the expression of PMN-related adhesion proteins, thus promoting the infiltration of PMN at the site of inflammation, enhancing vascular permeability and mucus secretion, and in turn triggering inflammatory responses such as edema.

Montelukast is a novel LTB receptor antagonist that can effectively block the binding of LTB to its receptors, thereby acting to antagonize inflammatory mediators. Preliminary studies have investigated the use of montelukast to treat bronchiolitis obliterans in a clinic setting [8, 9].

This study tests the hypotheses that LTB4 plays a part in post-transplantation bronchiolitis obliterans and that montelukast can reduce the degree of transplantation-related bronchiolitis obliterans.

## Methods

The protocol was reviewed and approved by the Ethics Committee of the First Affiliate Hospital of Zhejiang University.

The study protocol was based on donation from the F344 to the Lewis rat strain [10]. A population of pathogen-free male F344 rats and Lewis rats, 200 to 250 g in weight and 12 to 15 weeks in age, was kept and fed in laboratory conditions. After 1 week of adaptation to the environment, nine F344 rats and 18 Lewis rats with normal health and behavior were selected as donors and recipients, respectively, and randomly assigned into three groups: experimental, positive control, and blank control.

An allogeneic heterotopic tracheal transplantation model was established. The donors and recipients were anesthetized with 50 mg/kg pentobarbital sodium administered by intraperitoneal injection before surgery. After induction of anesthesia, the donors were fixed to the console in supine position. An incision was made at the top of the thyroid cartilage, and the surrounding tissues were bluntly separated to expose the trachea. The trachea was excised from the thyroid cartilage to the tracheal bifurcation, and the connective tissue attached to the adventitia was stripped away. Each specimen was rinsed with normal saline three times at 4 °C and cut into five cartilage rings lengthwise for use. After anesthesia, skin preparation, and disinfection, the recipients were dissected at the episternum with a 5-mm incision. A subcutaneous tunnel was dissected, and one piece of donor specimen was imbedded. The tissue was then stitched up and the skin disinfected [11, 12]. The recipients were returned to the cages of the corresponding groups after about 30 min of rewarming under incandescent light [13].

After 6 h of fasting and water deprivation, the rats received humane care for 28 days [14]. In the experimental group, the recipients were treated by gastric lavage with 3 mg/kg · d montelukast suspension, whereas in the positive control group and blank control group, gastric lavage was performed with 1 mg/kg · d dexamethasone and 1 mL/kg · d NS, respectively [15].

On Day 28 after operation, peripheral blood was drawn from the tail vein of the recipient rats, and the white blood cell count was taken [11]. The plasma was then separated by centrifugation, and the plasma LTB4 levels were measured by enzyme-linked immunosorbent assay.

The recipient rats were sacrificed on Day 28 after operation. The donor specimens were dislodged, fixed in 4% paraformaldehyde solution, paraffin-embedded, cut into 4-μm-thick sections, and stained by H-E and Masson for visual inspection of their organizational structure and fibrosis. The fibrosis scores of the grafts were evaluated. Regarding the cross-section of the trachea to be oval, the tracheal occlusion of each graft was calculated as the percentage of the cartilage ring that was affected by fibrous hyperplasia.

The experimental data were entered into a database using Microsoft Excel (Microsoft Corporation, Redmond, WA). All statistical calculations were performed using SPSS 20.0 software (IBM Corporation, Armonk, NY). The measurement data were compared using one-way analysis of variance, and the categorical data were compared using the chi-square test. A *P* value of less than 0.05 was considered to indicate statistical significance. Figures were drawn with GraphPad Prism 5.0 software (GraphPad Software Inc, La Jolla, CA).

## Results

The heterotopic tracheal transplantation model was successfully established. All operations were completed within 10 to 20 min. None of the 18 recipients suffered infection or died during the 28 days of the study.

**Fig. 1:**
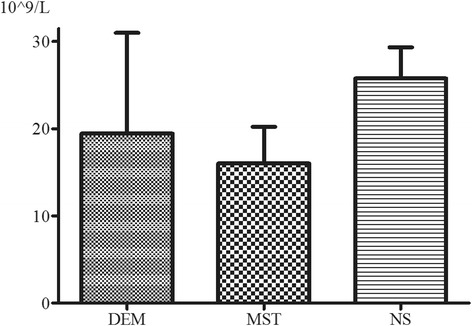
Peripheral white blood cell count on day 28 after operation. DEM, dexamethasone, positive control group; MST, montelukast, experimental group; NS, normal saline solution, blank control group. Columns and error bars represent mean with standard deviation. No statistically significant differences were found among the three groups

The peripheral white blood cell counts of the experimental group, positive control group, and blank control group were (16.0 ± 4.2) × 10^9^/L, (19.5 ± 11.6) × 10^9^/L, and (25.8 ± 3.6) × 10^9^/L (Fig. 1). One-way analysis of variance showed no statistical significance for these differences (*P* = 0.101).

**Fig. 2:**
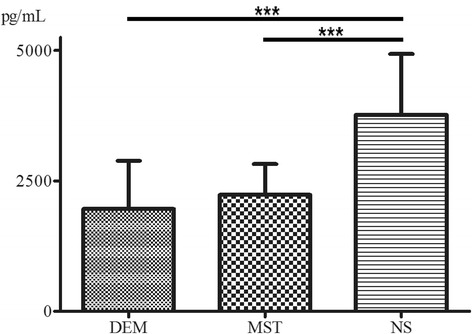
Results of enzyme-linked immunosorbent assay for LTB4 in peripheral blood on day 28 after operation. Statistically significant differences were found between the blank control group and the other groups

The concentrations of LTB4 in the peripheral blood of the experimental group, positive control group, and blank control group were 2230 ± 592 pg/mL, 1961 ± 922 pg/mL, and 3764 ± 1169 pg/mL (Fig. 2). The one-way analysis of variance and post hoc chi-square tests showed a statistical significance for the differences between the blank control group and each of the others (*P* = 0.009).

In the control group, the transplanted tracheas were infiltrated by interstitial cells, the bronchial wall was replaced by fibroplasia, and the lumen was significantly narrowed, resembling the histologic changes of bronchiolitis obliterans after clinical lung transplantation. In contrast, in the other groups, the transplanted tracheas were more complete in structure, without obvious mononuclear cell infiltration.

**Fig. 3:**
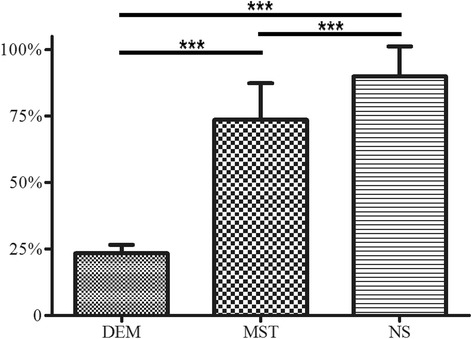
Tracheal occlusion of trachea on day 28 after operation. Statistically significant differences were found among the three groups

**Fig. 4:**
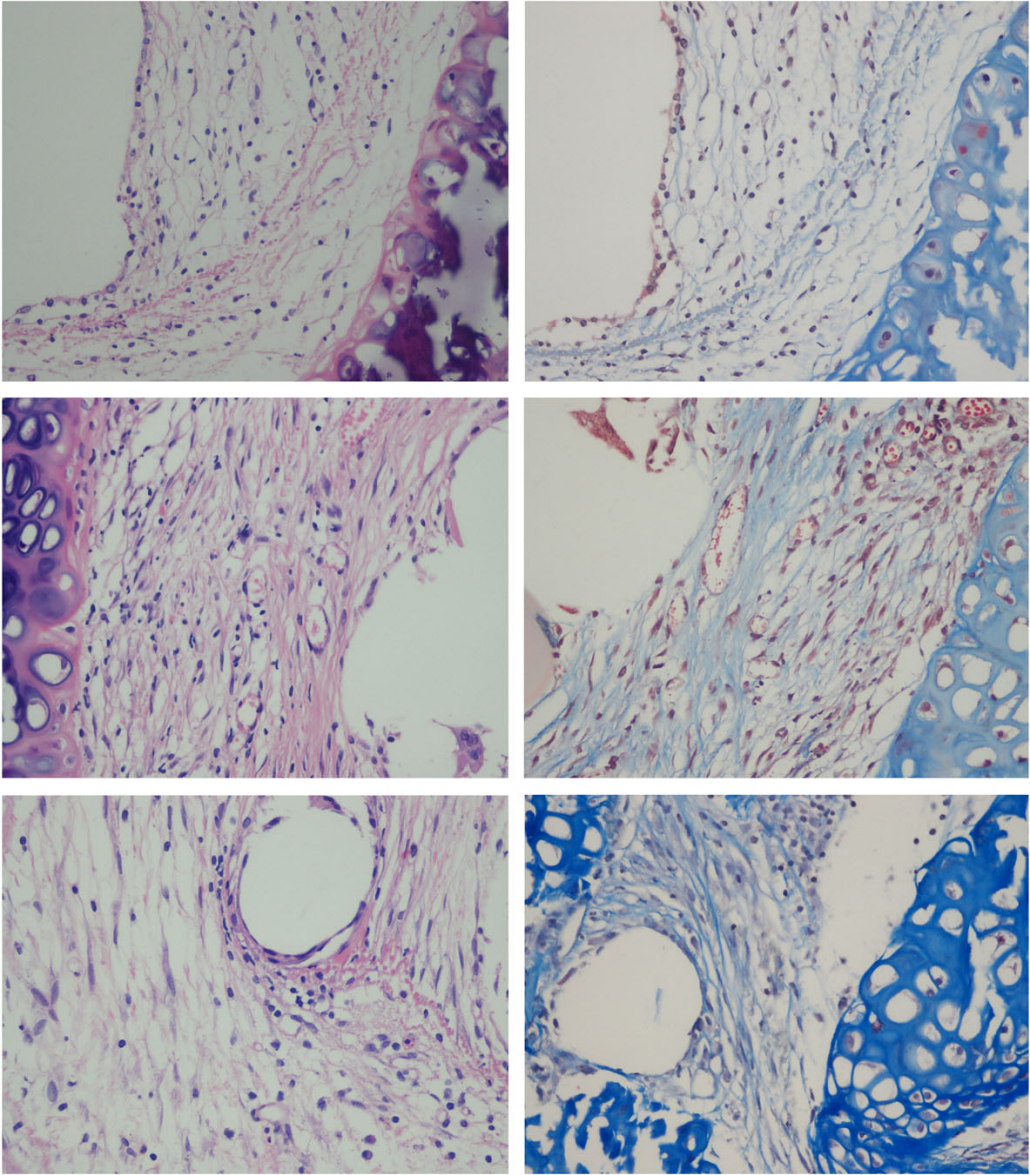
Tracheal occlusion and fiber hyperplasia on day 28 after operation. Left column: H-E staining, 400× magnification; right column: Masson staining, 400× magnification. Upper row: positive control group; middle: experimental group; lower: blank control group

In the blank control group, the transplanted tracheas showed obvious abnormalities under the microscope, including infiltration by inflammatory cells, active hyperplasia of the bronchial wall, and stenosis of the lumen, similar to the symptoms of bronchiolitis obliterans in clinical lung transplantation. The tracheal tissues were more complete in the other groups. The percentages of tracheal occlusion of the experimental group, positive control group, and blank control group were 73.6% ± 13.8%, 23.4% ± 3.2%, and 89.9% ± 11.3%, respectively (Fig. 3). The one-way analysis of variance and post hoc tests showed a statistical significance for the differences among all three groups (*P* = 0.000). The H-E-stained sections are shown in Fig. 4.

In the blank control group, the bronchial medial basal layer was thickened, and the smooth muscle showed structural disorder, with deep and continuous fiber hyperplasia. In the positive control group, the bronchial muscle layer was thinner than that in the blank control, and only a small amount of loosely arranged fibrous hyperplasia tissue was visible. The degree of fibrosis in the experimental group was between those of the two control groups. The Masson-stained sections are shown in Fig. 4.

## Discussion

Lung transplantation is the only effective clinical method to treat end-stage pulmonary diseases [16, 17]. Early postoperative survival rates continue to improve with the maturity of surgical techniques, the introduction of new generations of immunosuppressive agents, and the use of extracorporeal membrane oxygenation (ECMO) [18–20], but long-term survival is still subject to chronic complications such as bronchiolitis obliterans. Bronchiolitis obliterans has become the single greatest factor restricting long-term survival and quality of life after lung transplantation [21]. The pathogenesis of bronchiolitis obliterans, and thus the prevention and treatment strategy, are the central problems to be solved [22]. Thus, the establishment of a suitable model to mimic chronic rejection after clinical lung transplantation is critical to the study of this syndrome.

A 28-day period was adopted in this study because pathological changes have been found to worsen significantly over time. Studies of ischemia-reperfusion injury and acute rejection have conventionally used orthotopic lung transplantation models. However, in studies of chronic rejection and bronchiolitis obliterans, the demands of the surgical techniques and laboratory equipment, as well as the risk of graft rejection caused by necrosis without the intervention of immunosuppressive agents, must be considered. Moreover, some studies have suggested that this model is not typical of the pathological changes of bronchiolitis obliterans and has poor reproducibility [23]. Therefore, an ectopic tracheal transplantation model was selected for this study [24]. Its advantages include simple operation, good receptor tolerance, low risk of graft infection, and a short experimental period of 3 to 4 weeks. Furthermore, the pathologic changes of the bronchioles and terminal bronchioles are similar to those after clinical lung transplantation [25]. Steroid hormones are the preferred clinical treatment for bronchiolitis obliterans, especially in children, because they reliably inhibit inflammation and fibrosis. Thus, dexamethasone, a commonly used steroid hormone, was used in this study to model the state in which bronchiolitis obliterans is suppressed (the positive control group).

The models in this study also showed notable infiltration by inflammatory cells, mucosal thickening, disordered arrangement of smooth muscle, fibrous hyperplasia, and airway obstruction at day 28, which are characteristics of airway remodeling. These findings indicated that the heterotopic tracheal transplantation model had been successfully established.

LTB4 is a potent neutrophil chemoattractant and activator and a very important regulator of inflammation in vivo [26]. LTB4 can activate and aggregate a variety of inflammatory and immune effector cells [27]. It works on the cytokines released by T lymphocytes, such as IL-1, TNF-α, and matrix metalloproteinase-2, −3, and −9. LTB4 can induce airway inflammation by inhibiting the apoptosis of neutrophils and inducing their directional migration, thereby regulating the differentiation of immune cells and the expression of cytokines [28].

In this study, after 28 days, the concentration of LTB4 in peripheral blood was significantly lower in the experimental group than in the blank control group, whereas the tracheal occlusion rate of the experimental group was between those of the two control groups. This suggests that the LTB4 receptor antagonist montelukast can inhibit graft fibrosis to a certain extent and that LTB4 may be involved in the pathological process of chronic rejection after transplantation.

The proposal that LTB4 plays a role in chronic rejection after tracheal transplantation in rats was further supported by our study of the protective effect of montelukast on the trachea after heterotopic transplantation.

However, it is worth noting that tracheal transplantation is not completely equivalent to lung transplantation. The pathophysiological statuses of the graft and receptor are similar to but not exactly the same as those in the lung transplantation model. Therefore, the conclusions of this study require further experimental validation.

## Conclusions

In summary, this study demonstrates that LTB4 plays an important role in chronic rejection after tracheal transplantation in rats. Oral administration of montelukast reduces plasma LTB4 levels in rats and plays a preventive role against post-transplantation tracheal fibrosis. Although the specific pathways of montelukast remain to be uncovered by further study, the conclusions of this study provide a new conceptual basis for future research and treatment of chronic rejection after lung transplantation.

## Abbreviations

LTB4: Leukotriene B4
NS: Normal saline
ECMO: Extracorporeal membrane oxygenation
